# A Novel Method for Increasing the Numerousness of Biometrical Parameters Useful for Wildlife Management: Roe Deer Mandible as Bone Model

**DOI:** 10.3390/ani10030465

**Published:** 2020-03-11

**Authors:** Elena De Felice, Cesare Pacioni, Federico Maria Tardella, Cecilia Dall’Aglio, Antonio Palladino, Paola Scocco

**Affiliations:** 1 School of Biosciences and Veterinary Medicine, University of Camerino, Via Pontoni 5, 62032 Camerino, Italy; elena.defelice@unicam.it (E.D.F.); cesarepacioni@hotmail.com (C.P.); dtfederico.tardella@unicam.it (F.M.T.); paola.scocco@unicam.it (P.S.); 2 Department of Veterinary Medicine, University of Perugia, Via San Costanzo 4, 06126 Perugia, Italy; 3 CESMA-Center for metrological and advanced technological services, University of Naples Federico II, Cupa Nuova Cintia 21, 80146 Naples, Italy; a.palladino1986@gmail.com

**Keywords:** biometry, size analysis, shape analysis, wildlife management, roe deer

## Abstract

**Simple Summary:**

The wildlife expansion in the Italian Apennines caused a general development in hunting activities, together with the necessity of using biometry (size analysis) and geometric morphometry (shape analysis) as methods for monitoring the status of wildlife populations. Thus, in the last decades, study of the sizes and shapes of structures in wildlife populations has been extensively investigated. Biometric surveys and analysis of the resulting cranial and body data are now crucial in management decisions and new possibilities of improving datasets should be considered. Thus, we attempted to identify a conversion factor between shape and size analysis methods, using the mandible of adult roe deer as a bone model. The availability of this conversion factor enhances the numerousness of parameters into the classical biometric database, by means of the conversion of shape measures into size measures. Therefore, the relationship among biometric parameters, animal and environmental features can be better studied. The obtained data can be very useful to assess both wildlife population status and its management.

**Abstract:**

Study of dimensions (biometry) and shapes (geometric morphometry) of bone structures in ungulates is of extreme importance in wildlife population management. Unlike classical biometry, which involves the use of a caliper for measurements, geometric morphometry acquires, through software, a series of reference points (landmarks) from digital photos, providing a series of linear measures. A method to convert values obtained from the GeoGebra software into biometric measures is described. We took photos of 25 mandibles of adult roe deer and at the same time measured mandible length and teeth row length using a caliper. After image processing using GeoGebra, we calculated the conversion factor as the mean ratio between measures taken using GeoGebra and the caliper. The series of measurements, taken with two different methods (direct measurement using the caliper and conversion from GeoGebra output), showed a good degree of agreement. We used the conversion factor to obtain, from the GeoGebra database, four additional parameters of 50 mandibles. The analysis of variance showed that one parameter was significantly different between sexes (*p* = 0.04), demonstrating the usefulness of the measurement conversion. The conversion factor is helpful to improve classical biometric databases to better clarify the relationship between environment and wildlife status.

## 1. Introduction

Over the last few decades, the dimensions and shapes of structures in wildlife populations have been extensively investigated [1,2,3,4,5,6]. Biometric surveys and analysis of the resulting cranial and body data are now crucial in wildlife management decisions. The necessity of involving this kind of dataset in wildlife management has been particularly relevant in Italy, where the number of wild animals has considerably increased during the last decades [7], and where the procedures of measurement are nowadays standardized [8]. The expansion of wildlife populations [7] has, therefore, caused a general development in hunting activities, together with the necessity of using biometry (size analysis) and geometric morphometry (shape analysis) as methods to monitor the status of wildlife populations and suitable environments for different wildlife species [9,10,11,12]. The morphometric data analysis provides information on skeletal development and allows the identification of body measures useful as ecological indicators, permitting the formulation of better management strategies for wildlife [2]. Previous research demonstrated a relationship between biometric parameters (e.g., body mass, cranial and skeletal measures) and environmental features (e.g., climate, habitat quality, autumn-winter food availability) [2,5,12,13]. The monitoring of morphometric parameters can also be helpful for long-term studies on populations [2]; for example, the body mass of young roe deer is a good indicator of population productivity, while the body mass of adult animals responds to regional differences and to variations in population density and food availability [5,13]. Hence, new possibilities of improving datasets should be considered. 

Unlike biometry, which involves the use of a caliper for measurements [8], shape analysis acquires, through software, a series of reference points (landmarks) from digital photos, also providing a series of potential linear measurements, not taken in classic biometry. However, the linear measurements recorded by software like GeoGebra represent distances between Cartesian coordinates, evaluated on image projections and, therefore, they do not express the real values in the usual units of measurement. Thus, the calculation of a conversion factor between biometric measures and shape analysis-derived distances would allow the availability of many more parameters, which could be integrated in the classical biometric databases. These extra data could be correlated with environmental factors, in order to plan better management according to the territory carrying capacity [13].

In this research, we aimed to identify the possibility of obtaining a conversion factor, which would allow the acquisition of additional biometric measurements from shape analysis, in order to enhance the numerousness of biometric parameters. To reach this purpose we used the roe deer mandible, which is often used to evaluate the growth of ungulates in both size [14,15] and shape [16] analyses, as the bone model. Indeed, considering the available dataset, mandibular measurements are the most abundant among the cranial parameters. This is likely because the mandible is a bone structure that generally preserves its own characteristics during the post-shooting preparation process, making it a good candidate to look for new biometric parameters.

We tested this conversion factor by converting the measures of four additional mandibular parameters to highlight differences between sexes.

## 2. Materials and Methods

### 2.1. Method to Define the Conversion Factor

**Figure 1:**
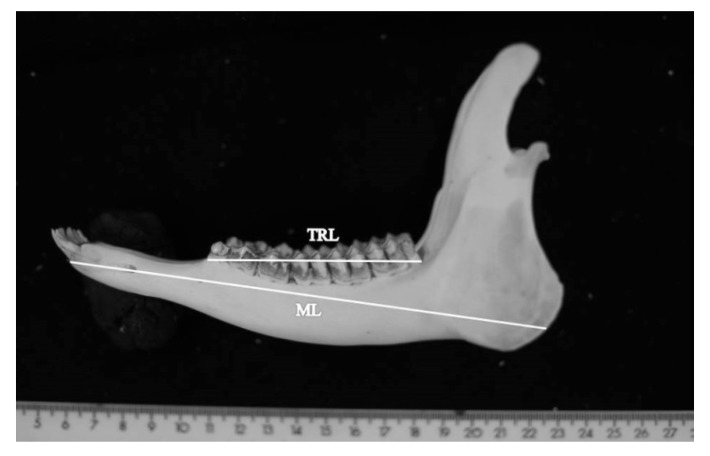
Photomacrography of the mandible with the two biometric parameters used: mandible length (ML) and teeth row length (TRL).

In order to find the conversion factor between the two measurement methods (caliper and GeoGebra), 25 mandibles (14 males and 11 females) pertaining to adult roe deer (*Capreolus capreolus* L.) (>11 months old), coming from the selective hunting activities in the ATC-MC2 Territorial Hunting Zone in Macerata Province (central Italy), were used. Attention was focused on two biometric parameters: mandible length (measured using the caliper, hereafter referred to as ML_C_, and derived from GeoGebra 5.0, hereafter ML_G_) and teeth row length (hereafter, TRL_C_ and TRL_G_) (Figure 1). 

To guarantee that the same points were taken into account in the two different measurement methods, the caliper measures were carried out while the photograph was taken. 

The conversion factor was calculated as the mean ratio between the two series of measures for mandible length (ML; i.e., Σ(ML_G*i*_/ML_C*i*_)/*n*), and teeth row length (TRL; i.e., Σ(TRL_G*i*_/TRL_C*i*_)/*n*) where *i* is the measure identity and *n* the total number of measures. Then, ML_G_ and TRL_G_ were transformed by dividing them by the conversion factor. This transformation ensured the comparability of the series of measures obtained by the two methods. The Levene’s test was performed to check the homogeneity of variances between ML_C_ and the transformed ML_G_ and between TRL_C_ and the transformed TRL_G_, using the leveneTest function (*car* R-package, version 3.0-6). The validity of the assumptions for linear regression between the two above-mentioned pairs of variables was ascertained using the gvlma function (*gvlma* R-package, version 1.0.0.3). To assess the degree of agreement between the two methods, the Bland–Altman regression [17,18,19] was run between ML_C_ and the transformed ML_G_ and between TRL_C_ and the transformed TRL_G_. The bias (average of the differences between measures), the upper and lower limits of agreement, and their confidence intervals (95%) were calculated and plotted using the blandr.display.and.draw function (*blandr* R-package, version 0.5.1). 

### 2.2. Conversion and Analysis of Additional Measures

**Figure 2:**
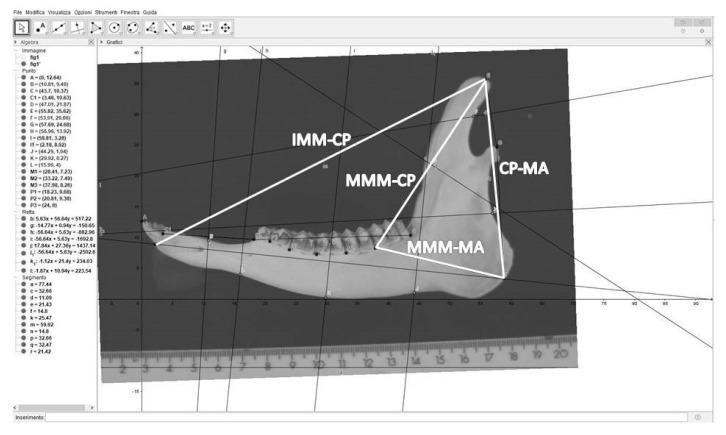
Image of a mandible, imported in the GeoGebra software, showing the four additional mandibular parameters, which were converted by means of the conversion factor. IMM-CP = distance from the mesial margin of the first incisor (IMM) to the coronoid process (CP); MMM-CP = distance from the mesial margin of the 3rd molar tooth (MMM) to the coronoid process; MMM-MA = distance from the mesial margin of the 3rd molar tooth to the angle of the mandible (MA); CP-MA = distance from the coronoid process to the angle of the mandible.

The conversion factor was used to convert measures of four additional mandibular parameters, taken using GeoGebra, from 50 mandibles sampled from adult roe deer (25 males and 25 females). The four parameters were (Figure 2):

- distance from the mesial margin of the first incisor (IMM) to the coronoid process (CP), hereafter IMM-CP;
- distance from the mesial margin of the 3rd molar tooth (MMM) to the coronoid process (CP), hereafter MMM-CP;
- distance from the coronoid process (CP) to the angle of the mandible (MA), hereafter CP-MA;
- distance from the mesial margin of the 3rd molar tooth (MMM) to the angle of the mandible (MA), hereafter MMM-MA.

These data, divided by sexes, were submitted to the Shapiro–Wilk test using the shapiro.test function (*stats* R-package, version 3.6.2) to ascertain the distribution normality, and to Levene’s test using the leveneTest function (*car* R-package), to assess the homogeneity of the variance. The null hypothesis of no effect of sex on each parameter was tested by the analysis of variance (ANOVA), using the aov function (*stats* R-package), when data met the assumptions required for parametric tests. When data did not meet these assumptions, the non-parametric robust ANOVA was performed, using the raov function (*Rfit* R-package, version 0.24.2). *P*-values were adjusted for multiple comparisons using the Holm correction. All the statistical analyses were run using R software (version 3.6.2, R Foundation for Statistical Computing, Vienna, Austria; http://www.R-project.org).

## 3. Results

The two series of mandibular measures taken by a caliper and GeoGebra are reported in Table S1 and data are summarized in Table 1.

The ratio between measurements belonging to both data series (ML_G_/ML_C_ and TRL_G_/TRL_C_) was quite constant (average of 0.040 ± 0.0003 SD in the former data series, and 0.040 ± 0.0004 in the latter), so that we assumed 0.04 as the searched conversion factor. 

**Figure 3:**
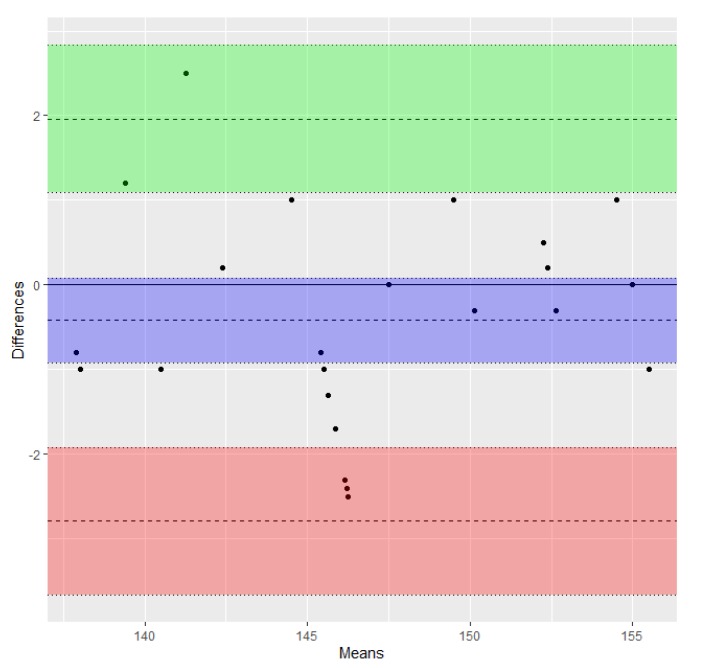
Bland–Altman plot of differences between ML measures obtained using the two methods (transformed ML_G_/ML_C_) vs. the mean of the two measures (data from Table 1). The bias or average of the differences (−0.42) and the lower and upper limits of agreement (−2.79 and 1.96) are represented as dashed lines. Lower and upper 95% confidence intervals of the bias (−0.92 to 0.09), lower limit of agreement (−3.66 to −1.93), and upper limit of agreement (1.09 to 2.83) are represented as dotted lines. Shaded areas include confidence interval limits for bias (blue) and agreement limits (green and red).

**Figure 4:**
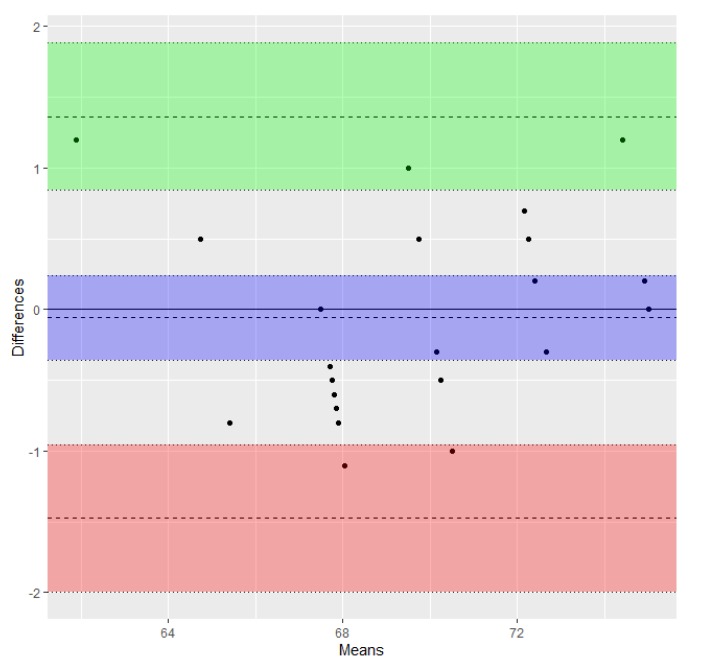
Bland–Altman plot of differences between TRL measures obtained using the two methods (transformed TRL_G_/TRL_C_) vs. the mean of the two measures (data from Table 1). The bias or average of the differences (−0.06) and the lower and upper limits of agreement (−1.48 and 1.36) are represented as dashed lines. Lower and upper 95% confidence intervals of the bias (−0.36 to 0.24), lower limit of agreement (−1.99 to −0.96), and upper limit of agreement (0.85 to 1.88) are represented as dotted lines. Shaded areas include confidence interval limits for bias (blue) and agreement limits (green and red).

The validity of this statement was demonstrated by the results of the Bland–Altman regression, which showed agreement between the two series of ML and TRL measures, taken using a caliper and the respective GeoGebra values transformed using the conversion factor (limits of agreement −2.79 to 1.96, and −1.48 to 1.36, respectively) as shown in Figure 3 and Figure 4. The limits were small enough to be confident that measures estimated with the new method can be used in the place of those taken with the caliper. Transformed values (tML_G_ and tTRL_G_) are shown in Table 1.

The mean values of the four additional parameters are summarized in Table 2. Among them MMM-MA showed significant differences between sexes (*p* = 0.04). 

## 4. Discussion

The collection of biometric data is extremely important in wildlife management. It allows to characterize the population, describing average trends and variability. In addition, a biometric database is useful to evaluate the overall performance of the population year by year and to estimate its health status. 

Moreover, the long-term monitoring and analysis of biometric measurements could represent a useful tool to complement the study of ungulate populations, as well as to give a broader insight into their environmental and interspecific interactions. It is, therefore, necessary to lay emphasis on the collection of biometric data in wildlife management and to have target parameters. 

We chose the roe deer mandible as a bone model because it is one of the first bones to complete its growth and therefore, its development is very sensitive to several environmental factors [5,6,12,20,21]. 

In this study, we found that the conversion factor allowed us to get additional parameters which were inserted into the classical biometric database and then analyzed to obtain information about the wildlife population status. These parameters can be correlated with a lot of different features such as age [1,13], sex [13,22,23], geographical features factors [6,24], population density [5], and trophic resource availability [13]. Moreover, the availability of this conversion factor can be useful in the acquisition of measures that could be missing from the database of measures obtained using a caliper due to, for example, a distraction or forgetfulness of the measurement collectors. It should be noted that the conversion factor must be calculated every time based on the measurement of at least one parameter recorded with the caliper at the same time that the photo is taken. 

We used the conversion factor to obtain and analyze four additional parameters, which are not taken in classic biometry. Our findings indicated that among the new parameters, MMM-MA showed significant differences between sexes. In this way, we demonstrated that a parameter, whose measure was not previously available, was influenced by sex.

In this study, we focused on the mandible, but the method can be extended to all cranial parameters or those of different bone structures. The extra data derived from software specific for shape analysis, thanks to the improvement of the biometric databases, can be useful in the applied study to help in management decisions, such as selective hunting activities [13]. While the roe deer mandibular biometric parameters collected for classical biometrical databases did not show any significant difference between sexes [13], this new method highlighted significant differences between sexes in the distance from the mesial marginal of the 3rd molar tooth to the angle of the mandible (MMM-MA).

Finally, this new method can enrich the existing classic biometric databases with extra data from shape analysis, when at least one size measurement is recorded at the moment of photo acquisition.

## Figures and Tables

**Table 1:** Mean and standard error of the mean (SEM) of mandible length and teeth row length values of twenty-five roe deer individuals (11 females and 14 males), measured using a caliper and derived from GeoGebra, and the estimated values after transformation of GeoGebra values using the conversion factor.

Sex	*N*	Measures Mean and SEM	MLc (mm)	TRLc (mm)	ML_G_	TRL_G_	tML_G_ (mm)	tTRL_G_ (mm)
Female	11	Mean	147.7	69.3	5.9	2.8	147.0	69.3
		SEM	1.7	0.9	0.1	0.0	1.8	1.0
Male	14	Mean	146.2	69.7	5.8	2.8	145.9	69.6
		SEM	1.3	0.9	0.1	0.0	1.3	0.9

ML_C_ = caliper mandibular Length; TRL_C_ = caliper teeth row length; ML_G_ = GeoGebra mandibular length; TRL_G_ = GeoGebra teeth row length; tML_G_ = transformed GeoGebra mandibular length; tTRL_G_ = transformed GeoGebra teeth row length.

**Table 2:** Mean ± standard deviation (SD) and significance values (*p*) of the comparisons between sexes of the parameter values (mm) estimated using the conversion factor. *P*-values have been adjusted for multiple comparisons.

Parameters ^1^	Male (Mean ± SD)	Female (Mean ± SD)	*p*
IMM-CP	159.5 ± 7.4	156.4 ± 15.4	0.612
MMM-CP	86.5 ± 3.9	85.7 ± 5.2	0.692
CP-MA	85.8 ± 5.7	84.3 ± 6.3	0.692
MMM-MA	52.7 ± 6.4	54.3 ± 5.3	0.040

IMM-CP = distance from the mesial margin of the first incisor (IMM) to the coronoid process (CP); MMM-CP = distance from the mesial margin of the 3rd molar tooth (MMM) to the coronoid process; MMM-MA = distance from the mesial margin of the 3rd molar tooth to the angle of the mandible (MA); CP-MA = distance from the coronoid process to the angle of the mandible.

## Supplementary Materials

The following are available online at https://www.mdpi.com/2076-2615/10/3/465/s1, Table S1: Series of mandibular measures of mandible length and teeth row length values of twenty-five roe deer individuals, measured using a caliper and derived from GeoGebra, and the estimated values after transformation of GeoGebra values using the conversion factor.
