# Supplementary material for: A Novel Method for Increasing the Numerousness of Biometrical Parameters Useful for Wildlife Management: Roe Deer Mandible as Bone Model
Source: Animals (Basel). 2020 Mar 11;10(3):465. doi: 10.3390/ani10030465 (PMC7142537; doi:10.3390/ani10030465)
Supplement: Supplementary file 1 [file animals-10-00465-s001.pdf]

A novel method for increasing the numerousness of biometrical parameters useful for wildlife management: roe deer mandible as bone model

Elena De Felice, Cesare Pacioni, Federico M. Tardella, Cecilia Dall'Aglio, Antonio Palladino, Paola Scocco

Table S1. Series of mandibular measures of mandible length and teeth row length values of twenty-five roe deer individuals, measured using a caliper and derived from GeoGebra, and the estimated values after transformation of GeoGebra values using the conversion factor.

Sex	ML_C (mm)	TRL_C (mm)	ML_G	TRL_G	tML_G (mm)	$tTRL_G$ (mm)
f	147.4	68.2	5.8	2.7	145.0	67.5
f	154.0	74.8	6.2	3.0	155.0	75.0
f	146.3	67.9	5.8	2.7	145.0	67.5
f	152.8	72.3	6.1	2.9	152.5	72.5
f	138.8	64.5	5.6	2.6	140.0	65.0
f	145.8	69.0	5.8	2.8	145.0	70.0
f	145.8	71.0	5.8	2.8	145.0	70.0
f	138.3	65.8	5.5	2.6	137.5	65.0
f	152.3	69.0	6.1	2.8	152.5	70.0
f	156.0	72.0	6.2	2.9	155.0	72.5
f	146.7	68.1	5.8	2.7	145.0	67.5
m	152.0	70.5	6.1	2.8	152.5	70.0
m	147.5	68.0	5.9	2.7	147.5	67.5
m	155.0	75.0	6.2	3.0	155.0	75.0
m	142.3	61.3	5.7	2.5	142.5	62.5
m	150.3	70.3	6.0	2.8	150.0	70.0
m	144.0	73.8	5.8	3.0	145.0	75.0
m	145.8	68.3	5.8	2.7	145.0	67.5
m	149.0	71.8	6.0	2.9	150.0	72.5
m	141.0	70.3	5.6	2.8	140.0	70.0
m	138.5	69.5	5.5	2.8	137.5	70.0
m	146.0	72.8	5.8	2.9	145.0	72.5
m	147.5	68.6	5.8	2.7	145.0	67.5
m	147.3	68.6	5.8	2.7	145.0	67.5
m	140.0	67.5	5.7	2.7	142.5	67.5

f = female; m = male; ML_C = caliper mandibular length; TRL_C = caliper teeth row length; ML_G = GeoGebra mandibular length; TRL_G = GeoGebra teeth row length; tML_G = transformed GeoGebra mandibular length; $tTRL_G$ = transformed GeoGebra teeth row length
